# Transactions of Medical Society, West Virginia

**Published:** 1879-01

**Authors:** 


*Transactions of the Medical Society of the State of West Virginia*, 1878.

The above Volume is on our table and presents a bright and attractive appearance.

We should judge after an examination of the Proceedings and of the various papers presented, that our West Virginia brethren had an exceptionably fine meeting at Weston.

The lengthiest article is the one entitled, Continued "Report on Surgical Cases" by Dr. John Frissell, of Wheeling. It is a plain straightforward narration of cases occurring in the author's practice. Such a paper is an absolute and valuable acquisition to the Profession, being the conclusions arrived at by a conscientious and pains-taking Surgeon, derived from a practice extending over a period of forty years.

We especially noted the cases of Traumatic Aneurism; in this class of distressing injuries the author has had considerable experience, giving no less than eleven illustrative cases, eight of the axillary, one of the femoral, one of the popliteal and one of the facial arteries.

Case 114 is one of an amputation of the arm, where an axillary aneurism existed in connection with injury of the axillary nerves and veins.

The Doctor's rule to amputate in such cases is one founded on good Surgery. We feel sure that many lives will be spared should it be generally adopted by the profession.

There has been some bad blundering in numbering the cases, as we find case 114, or at least the principal part of it given under the caption of cases 115, 116, 117 and 118.

A contribution on Psychological Medicine, by Dr. Camden, of Weston, is quite readable. Some curious details are given of the old methods of treating insanity which contrast very strikingly with the system now pursued. Speaking of the immense strides that have been made in psychological medicines, the Doctor enthusiastically predicts that we will soon be able to "elucidate and demonstrate the real essence of mind itself—that subtle, invisible something so nearly allied to Deity." We are afraid the Doctor is mistaken. Beranger, the French poet, asks:

> Was ever such an ass as that,
> Who hoped, by slicing mutton fat,
> And pulling candlesticks to pieces,
> To tell why *Light* should spring from *Greases?*
> Yes—one—that still more precious fool,
> Who, in the anatomic school,
> Expected, with dissecting knife,
> To learn from *Death* the laws of *Life.*

There are a number of other papers alike creditable to the Society and to their authors. "The Diagnostic Value of Symptoms," by Dr. John Huff, is full of good practical common sense, as is also a paper on Puerperal Insanity, by Dr. A. H. Runot. The paper entitled "Concerning the Dressing of Wounds" contains much useful matter in reference to Prof. Lister's antiseptic treatment; the author, Dr. W. H. Sharp, expressly states that his paper is to a great extent a compilation, but we are afraid that the general slip-shoddings of composition pervading the whole article will induce most seekers for information to go to head quarters.

Pres. McSherry's Address and the paper of Dr. M. S. Hall treat essentially on the same subject—public hygiene. We trust they will serve to help bring about the establishment of a State Board of Health in that young and far distant State.

The Vol. also contains reports of two cases of Extra Uterine Pregnancy, by two different authors, Drs. Brownfield and Boyd, both of which are interesting.

